# Editorials

**Published:** 1911-05

**Authors:** 


### EDITORIALS

The Business Office of the *Journal-Record* is 1 1-2 to 5 1-2 South Broad Street.
The Editorial Office is 1014-15 Century Building.
Address all Business Communications to *Journal-Record of Medicine*, 1 1-2 to 5 1-2 South Broad Street.
Make remittances payable *to The Journal-Record of Medicine.*
On matters pertaining to the Editorial and Original Communications, address Edgar G. Ballenger, M. D., Atlanta, Ga.
Reprints of Original Articles will be furnished at cost price. Requests for the same should always be made in THE MANUSCRIPT.
We will present, postpaid, on request, to each contributor of an original article, twenty (20) marked copies of *The Journal-Record of Medicine* containing such article.

### DEATH OF DR. T. C. LONGINO.

It is with profound regret that we record the death of Dr. Thomas C. Longino, who was one of Atlanta's most popular and lovable young physicians. Dr. Longino had been sick for several months and his death was not unexpected. He was 37 years of age and had been very successful in his practice.

Shortly after leaving school, he enlisted in the army, serving during the campaign of the Spanish-American war as assistant surgeon. Following the close of the war, he removed to Galveston, Texas, and was present during the tidal wave which destroyed that city. His valiant work served to save scores of injured, and while working among the victims of this flood he met his wife, Miss Morton Campbell, one of Galveston's prominent young women, a daughter of Mr. and Mrs. J. S. Campbell. Later he removed to Atlanta, where he became actively engaged in the practice of medicine.

---

### BETTER MILK FOR ATLANTA BABIES.

A commendable movement by the Fulton County Medical Society is the appointment of a committee which is to make an effort to arrange for "certified milk" in Atlanta, so that babies may be supplied with milk not containing an excess of bacteria, and pus. There is no reason why such an effort should not be eminently satisfactory, as we feel sure the city board of health will lend its assistance, and by the payment of a somewhat higher rate dairymen who take extra care in the selection of their cows, and in the handling of milk should make the sale of such milk profitable.

As the hot weather approaches the growth of bacteria in the milk increases and the death in bottle-fed babies assumes an immediate increase. Many innocent lives are lost annually through inability to procure clean, wholesome milk, and through the carelessness and ignorance in the handling of it by the mothers in the homes. It is not sufficent that good milk be supplied, but it should be cared for in the homes so that the growth of bacteria is kept to the minimum. The average refrigerator does not keep milk as cold as it should be kept A useful expedient which will save nearly enough ice during one season to pay for itself and save more than enough babies to pay for the ice, is the so-called "fireless cooker." It mantains a very low temperature on account of the vacuum instead of packing, etc., to keep out the heat. It is small and can be used solely for the babies' milk.

---

### COMMENCEMENT OF ATLANTA MEDICAL COLLEGES.

After a very successful term as regards attendance and a commendable one as regards the increase in the preliminary education required, the Atlanta College of Physicians and Surgeons and the Atlanta School of Medicine have held ther commencement and graduated 95 young men. It is with much pleasure that we note an improvemnt in the entrance requirements and in increase in the medical training now required before the degree of M.D. is awarded, and we hope in the future to see a greater improvement—especially in the practical work; this last remark applies to nearly all medical colleges. The following were graduated from the Atanta College of Physicians and Surgeons:

From the state of Georgia, H. M. Belflower, W. E. Benson, F. Bird. L. L. Blair, B. R. Russell, T C. Clodfelter, W. L. Cousins, L. P. Daley, A. C. Dorminy, W. H. Dyer, H. T. Exley, R. M. Exley, C. B. Fluker, W. M. Folks, C. B. Greer, C. Griffin, R. D. Harden, G. W. Kelley, E. H. Lamb, J. C. McDougall, E. A. Moody, C. H. Paine, D. Payne, J. E. Pounds, A. J. Rodgers, A Schwartz, G. Selby, T. H. Smith, W. K. Swann, W. E. omasson, W. C Troutman. From Alabama, H. W. Birdsong, L. E. Brawen, L. E. Morton, E. W. Wheelis; T. H. Bradson of North Carolina, H. L. Bryans of Florida, W. T. Head of North Carolina, E. W. Holloway of Florida, J. C. Jones of Mississippi E. H. McRae of Florida, G. C. Pruitt of South Carolina, S. L. Turner of Florida, W. J. Vinson of Florida, J. Wallace of South Carolina.

Graduates from the Atlanta School ot Medicine were:

E. R. Brown, A. H. Bunce, H. V. Collins, J. G. DeVane, R. H. Eznor, O. F. Harper, G. R. Heaton, C. R. Henry, T. B. King, S. A. Kirkland, W. J. Lancaster, J. W. Landham, D. R. Longino, L. L. Lundy, S. W. Martin, J. I. Matthews, L. Neville, A. L. Prince, W. F. Reavis, J. A. Redfern, B. Rosenbloom, A. R. Rozar, E. E. Sapp, W. W. Sessions, J. M. Smith, M. W. Spearman, G. L. Thompson, C. M. Tyre, B. H. Wagnon, W. F. Wells, W. C. Williams, of Georgia; H. E. Evans, W. R. Grady, A. R. Ladner, T. J. Smith, of Mississippi; W. B. Henderson, J. W. Landham, R. B. McCann, M. C. Pruitt, of Alabama; E. V. Keller, of Missouri; L. F. Moralles, Cuba; E. Peters, W. H. Pickett, Florida; F. F. Rougon, Louisiana; W. A. Travena, Tennessee; R. F. Warren, North Carolina.

